# Treatment of Coral Reef Aorta by Endovascular VIABAHN VBX Balloon-Expandable Stent-Graft Placement

**DOI:** 10.3400/avd.cr.20-00168

**Published:** 2021-09-25

**Authors:** Kaoru Myouchin, Katsutoshi Takayama, Takeshi Wada, Hidehiko Taguchi, Toshihiro Tanaka, Kimihiko Kichikawa

**Affiliations:** 1 Department of Radiology and Interventional Radiology Center, Nara Medical University, Kashihara, Nara, Japan; 2 Department of Interventional Neuroradiology/Radiology Kouseikai Takai Hospital, Kashihara, Nara, Japan

**Keywords:** coral reef aorta, VIABAHN VBX, pressure wire

## Abstract

Coral reef aorta (CRA) has been described as a rare disease characterized by the presence of dense calcifications of the aorta. In this study, we report on two patients with CRA caused by intermittent claudication (IC) who underwent endovascular VIABAHN VBX balloon-expandable stent-graft (VVBX) placement. Both patients underwent successful endovascular VVBX placement via transfemoral artery approach, and hemostasis was achieved via vascular closure device. Their symptoms were observed to disappear completely after treatment, and they were discharged without serious adverse events. No symptoms were noted at 1.5-year and 1-year follow-up.

## Introduction

Coral reef aorta (CRA) has been described as dense calcifications of the aorta, which may result in ischemia of the lower limbs and the viscera and renovascular hypertension (HT). Endarterectomy, angioplasty, stent placement, and stent-graft placement have already been identified as the treatments for CRA. The VVBX (W. L. Gore & Associates, Flagstaff, AZ, USA) then became available for use in iliac occlusive disease treatment from July 2018 in Japan. To the best of our knowledge, there has been no report on CRA being treated with VVBX. Thus, in this study, we report on two patients with CRA who underwent endovascular treatment with VVBX. The approval of the off-label use of VIABAHN VBX for aortic stenosis was obtained from the institutional review board at our hospital; moreover, the two patients also provided consent for the publication of this report.

## Case Reports

Case 1: An 82-year-old woman was admitted to our service with intermittent claudication (IC). She had a history of HT and dyslipidemia (DL). The ankle-brachial index (ABI) was decreased to 0.6 on the right and 0.8 on the left.

**Figure 1:**
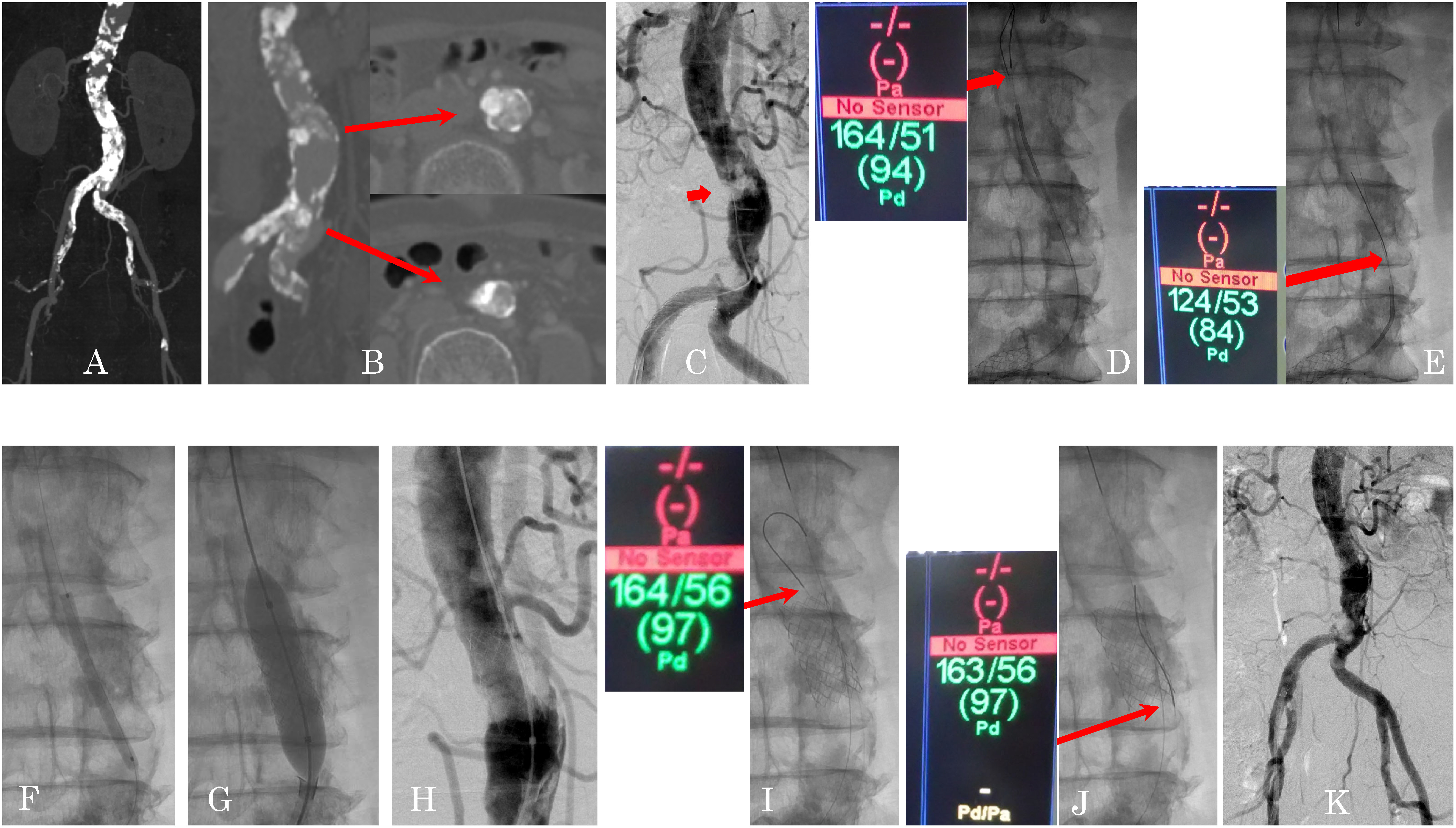
Fig. 1 Endovascular treatment for coral reef aorta with VIABAHN VBX (Case 1).

Computed tomography (CT) angiography showed two stenotic lesions of the infrarenal aorta, just distal to the renal artery and just proximal to the iliac bifurcation with calcification (**Figs. 1A** and **1B**).

(**A**) Computed tomography (CT) angiography (maximum intensity projection). (**B**) CT angiography (multiplanar reconstruction). CT angiography shows two stenotic lesions of the infrarenal aorta, just distal to the renal artery and just proximal to the iliac bifurcation with heavy calcification (arrows). (**C**) Pelvic angiography shows severe stenosis of the infrarenal aorta just distal to the renal artery (arrow) and moderate stenosis just proximal to the iliac bifurcation with heavy calcification. (**D**, **E**) (Live view and photograph) (**D**) distal, (**E**) proximal. The systolic pressure gradient is 40 mmHg at the stenotic lesion of the infrarenal aorta just distal to the renal artery using a pressure wire (arrows). (**F**) Pre-dilation is performed using a balloon catheter (3 mm×40 mm; JADE). (**G**) The VIABAHN VBX balloon-expandable stent-graft (10 mm×29 mm) is placed and inflated to the stenotic lesion at the infrarenal aorta just distal to the renal artery. (**H**) Digital subtraction angiography confirms good vascular dilation at the stenotic site, with no obvious aortic dissection. (**I**, **J**) (Live view and photograph) (**I**) distal, (**J**) proximal. The systolic pressure gradient is 1 mmHg at the stenotic lesion of the infrarenal aorta just distal to the renal artery by pressure wire (arrows). (**K**) Final angiography shows proper position of the VIABAHN VBX balloon-expandable stent-graft and good run-off.

Percutaneous cannulation of the left radial artery was then performed, followed by an insertion of a 0.025 inch attached wire, advancement of a 4 Fr sheath, and insertion of a pigtail catheter just proximal to the stenotic lesion. An aortogram was then obtained (**Figs. 1C**). Next, percutaneous cannulation of the right common femoral artery was performed, followed by insertion of a 0.035 inch attached wire and advancement of a 5 Fr sheath in the proximal right common iliac artery; attached wire was then changed to 0.035 inch Amplatz wire (Boston Scientific, Natick, MA, USA). A marker 8 Fr sheath (Cordis Endovascular, Miami Lakes, FL, USA) was advanced over the wire and placed in the proximal right common iliac artery. Then, a 0.014 inch Cruise wire (ASAHI INTECC, Aichi, Japan) was taken to cross the stenotic lesion, and Teleport OD 1.5 Fr microcatheter (OrbusNeich, Hong Kong) was advanced over the wire. Cruise wire was exchanged to 0.014 inch Aguru wire (Boston Scientific, Natick, MA, USA). Pre-dilation was performed using a balloon catheter (3 mm×40 mm; JADE, OrbusNeich, Hong Kong), and Vertebral tempo 4 Fr (Cordis Endovascular, Miami Lakes, FL, USA) was then advanced over the wire. Aguru wire was exchanged to 0.035 inch Amplatz wire, and a marker 8 Fr sheath was advanced over the wire and placed in the aorta and proximal stenotic lesion. A VVBX was planned to be placed for the stenosis of the infrarenal aorta if there was a significant systolic pressure gradient (PG). The PG was measured between the proximal and the distal two stenotic lesions using a pressure wire (PressureWire Certus; St. Jude Medical, Inc., St. Paul, MN, USA). The PG was 40 mmHg at the lesion distal to renal (**Figs. 1D** and **1E**), and the PG was only 8 mmHg at the lesion proximal to iliac. Therefore, it was decided to treat the lesion distal to renal artery. Then, intravascular ultrasound (IVUS) was performed. IVUS showed minimum lesion diameter (MLD) was 1.3×3.3 mm. Lumen of the aorta, distal to renal artery stenotic lesion, was 11.8×14.7 mm.

A VVBX (10 mm) was placed to the lesion distal to renal via an 8 Fr sheath (**Fig. 1G**). The lesion distal to renal was dilated appropriately on digital subtraction angiography (DSA) (**Figs. 1H** and **1K**); meanwhile, IVUS showed MLD was 8.0×8.5 mm, and the PG at the lesion distal to renal artery decreased to 1 mmHg (**Figs. 1I** and **1J**). Hemostasis was achieved with a 7 Fr EXOSEAL hemostatic device (Exo) (Cordis Corporation, Miami Lakes, FL, USA). The postoperative course was uneventful, and her symptoms completely disappeared. The next day, the ABI was increased to above 1.0. The patient was discharged without any complication 1 week later. Moreover, 1.5 years after the treatment, the patient remains asymptomatic, and her ABI also shows normal range.

**Figure 2:**
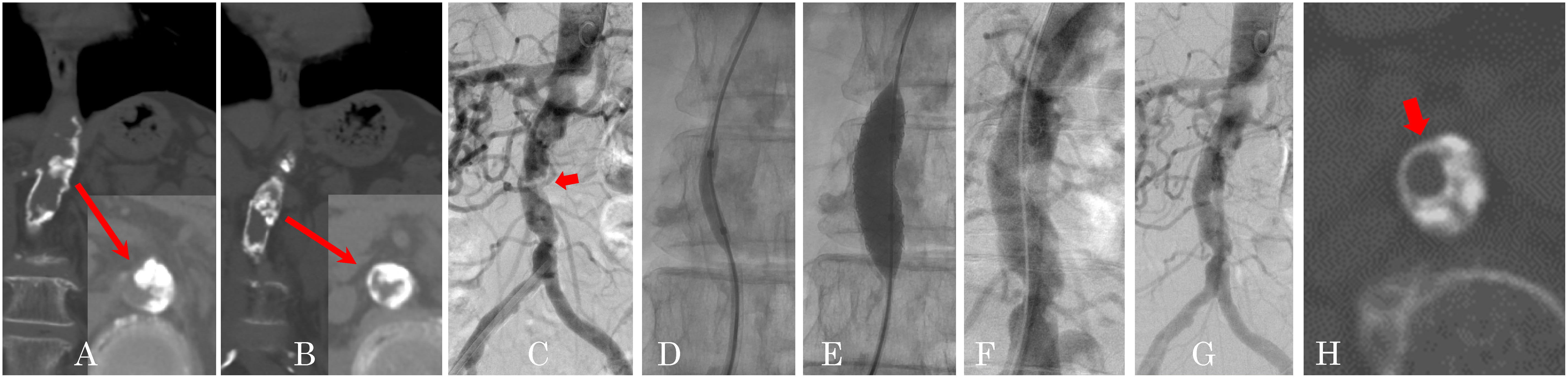
Fig. 2 Endovascular treatment for coral reef aorta with VIABAHN VBX (Case 2).

Case 2: An 84-year-old woman was admitted to our service with a 10-year history of IC. She had a history of DL. The ABI was decreased to 0.6 on the right and 0.7 on the left. There was evidence of renal impairment. Magnetic resonance angiography and plain CT showed two stenotic lesions of the infrarenal aorta with calcification (**Figs. 2A** and **2B**). Percutaneous cannulation of the left radial artery was then performed, followed by insertion of a 0.025 inch attached wire, advancement of a 4 Fr sheath, and insertion of a pigtail catheter to just proximal stenotic lesion. An aortogram was obtained. Next, percutaneous cannulation of the right common femoral artery was performed, followed by insertion of a 0.035 inch attached wire, advancement of a 5 Fr sheath in the proximal right common iliac artery; attached wire was then exchanged to 0.035 inch Amplatz wire, and a marker 7 Fr sheath was advanced over the wire and placed in the proximal right common iliac artery. Then, 0.014 inch GT wire (TERUMO CLINICAL SUPPLY CO., Gifu, Japan) was taken to cross the stenotic lesion, and Teleport OD 1.5 Fr microcatheter was advanced over the wire. GT wire was exchanged to 0.014 inch Aguru wire. Pre-dilation is performed using a balloon catheter (4 mm×20 mm; Mustang, Boston Scientific, Natick, MA, USA), and Vertebral tempo 4 Fr was advanced over the wire. Aguru wire was exchanged to 0.035 inch Amplatz wire; a marker 7 Fr sheath was advanced over the wire and placed in the aorta and proximal stenotic lesion.

(**A**, **B**) Plain computed tomography (CT) (multiplanar reconstruction). (**A**) just distal to the renal artery, (**B**) just proximal to the iliac bifurcation. Plain CT shows two stenotic lesions of the infrarenal aorta, just distal to the renal artery and just proximal to the iliac bifurcation with heavy calcification (arrows). (**C**) Pelvic angiography shows moderate stenosis of the infrarenal aorta, just distal to the renal artery and severe stenosis just proximal to the iliac bifurcation with heavy calcification (arrow). (**D**) Pre-dilation is performed using a balloon catheter (4 mm×20 mm; Mustang). (**E**) The VIABAHN VBX balloon-expandable stent-graft (8 mm×39 mm) is placed and inflated to the stenotic lesion at the infrarenal aorta just proximal to the iliac bifurcation. (**F**) Digital subtraction angiography shows good vascular dilation at the stenotic site, with no obvious aortic dissection. (**G**) Final angiography displays proper position of the VIABAHN VBX balloon-expandable stent-graft and good run-off. (**H**) Plain CT (the next day) shows good dilatation of the infrarenal aorta, just proximal to the iliac bifurcation with heavy calcification (arrow).

A VVBX was planned to be placed to the stenotic lesions if a significant PG were present. The PG was measured from the proximal to the distal two stenotic lesions using a diagnostic catheter. There was no PG (0 mmHg) at the proximal stenosis. On the other hand, at the distal stenosis, the PG was 49 mmHg at the stenotic lesion. Therefore, it was decided to treat the distal aortic stenosis (**Fig. 2C**). In this case, IVUS before treatment could not be performed due to a mechanical problem. A VVBX (8 mm) was then placed at the distal stenosis via a 7 Fr sheath (**Figs. 2D** and **2E**). The stenotic lesion was dilated appropriately on DSA (**Figs. 2F** and **2G**), and IVUS after stent placement showed MLD was 7.6×8.4 mm. And the PG at the stenotic lesion decreased to 7 mmHg. Hemostasis was achieved via a 7 Fr Exo. The postoperative course was uneventful, and her symptom completely disappeared. The next day, the ABI increased to above 1.0. The patient was discharged without any complication 1 week later. One year after treatment, the patient remains to be asymptomatic, and her ABI also shows normal range.

## Discussion

Back in 1984, Qvarfordt et al. were first to report on female patients with heavily calcified CRAs.^1)^ In a report by Grotemeyer et al.,^2)^ 70 patients with CRA were reported. The risk factors for atherosclerosis were noted. Patients with CRA had various manifestations, including renovascular HT, IC, and chronic visceral ischemia.

Traditionally, endarterectomy and angioplasty have been identified as the mainstay treatments for localized aortic atherosclerosis. In fact, Grotemeyer et al.^2)^ reported that 69 patients with aortoiliac calcification disease underwent endarterectomy. Mortality rate was at 11.6%, while morbidity rate was noted to be at 30.4% within 30 days after endarterectomy. In a study by Inahara et al.^3)^ as regards operative therapy, 59 patients with aortic atherosclerosis underwent endarterectomy. Two patients (3.7%) reportedly died within 30 days after endarterectomy. Given these reports, endarterectomy seems to be not the first option in treating CRA.

On the other hand, in another study by Vries et al.,^4)^ 69 patients with aortic atherosclerosis reportedly underwent angioplasty. The initial endovascular treatment succeeded in 68 patients. No procedure-related deaths occurred. However, in heavily calcified aortic stenosis, there seems to be a risk of vessel rupture. In fact, Chung et al.^5)^ reported that during procedure of endovascular treatment with stent-graft, for CRA, aortic rupture occurred. In this case, stent-graft was placed on the rupture point to resolve the leak.

Stent-grafts may become an alternative treatment, because it seems that there is a few risk of vessel rupture different from that of a bare stent. Chung et al. also recommend the use of covered instead of bare metal stents for cases involving severe, high-grade, atherosclerotic lesions, such as “coral reef” lesions, in which there is a high potential for aortic rupture.

In another study performed by Holfeld et al.,^6)^ CRA was treated with a GORE TAG (W. L. Gore & Associates, Inc., Flagstaff, AZ, USA), wherein it was deemed successful without any complications. Configurations of TAG available for use are compatible for 0.035 inch over the wire. Stent diameter is from 21 mm via a 18 Fr sheath to 45 mm via a 24 Fr sheath, and stent length is around 10, 15, and 20 cm.

VVBX endoprosthesis used in these cases has been noted to be made up of self-governing stainless steel rings attached via fluoropolymer graft material. Configurations available for use are all 0.035 inch over the wire configurations with 80 or 135 cm catheter lengths; stent diameter is under 8 mm via a 7 Fr sheath and 9 and 10 mm via an 8 Fr sheath; in addition, VVBX can be expanded until 13 mm using a balloon.

As VVBX is significantly smaller than TAG and has more strong radial force than TAG, VVBX seems to be more suitable for treating for CRA.

There has been a report by Bismuth et al.^7)^ that 134 patients with 213 iliac lesions were treated with VVBX. In total, 234 devices were implanted in 213 lesions with 100% technical success. At 9 months, 3 (2.3%) of 132 patients suffered from a major adverse event (3 target lesion revascularizations) related to the primary endpoint. At 9 months, there were no device-related adverse events. However, there are three major differences between the TAG and the VVBX. The first point is that the TAG has a much larger diameter than the VVBX; in fact, TAG that covered stent placement needs a 20 Fr sheath or a 22 Fr sheath in these cases; further, surgical vascular repair is needed for hemostasis after a transfemoral vascular procedure. On the other hand, since the VVBX can be placed via a 7 Fr or an 8 Fr sheath, surgical repair is not necessary. In fact, in both of the present cases, hemostasis was achieved with the Exo without any complications. The second point is that TAG is much weaker with respect to radial force than VVBX. CRA seems to be much harder than a conventional stenosis. Therefore, the VVBX seems to be appropriate for CRA. The third point is that precise stent placement is much easier with the VVBX than with the TAG, because the VVBX is a balloon-expandable stent. To the best of our knowledge, there has been no report regarding the treatment of CRA using VVBX. Based on the several points mentioned before, the VVBX is a more suitable stent for treatment for CRA.

As per the findings of Johnson et al.,^8)^ it was showed that there was no significant difference regarding phasic pressure assessment between the diagnostic catheter and the pressure wire by recording the PG of coronary and valvular stenosis. Therefore, we measured the PG using the pressure wire or the diagnostic catheter, and only the stenoses with a PG were treated. In fact, in both cases, the ABI and symptoms improved after treatment. Therefore, measuring the PG at a stenotic lesion seems be useful for making decisions regarding treatment strategy.

## Conclusion

VVBX placement was deemed beneficial in treating two CRA cases.

## Additional Note

The study follows the ethical standards of the institution.
